# *AtPADRE13* Negatively Regulates Salt Stress Tolerance in *Arabidopsis thaliana*

**DOI:** 10.3390/plants14101514

**Published:** 2025-05-19

**Authors:** Ziru Chang, Xiaona Tian, Xiaocui Niu, Meiting Bai, Wei Bai, Ruigang Wang, Guojing Li, Qi Yang

**Affiliations:** 1 Key Laboratory of Plants Adversity Adaptation and Genetic Improvement in Cold and Arid Regions of Inner Mongolia, Inner Mongolia Agricultural University, Hohhot 010018, China; cx15047824868@163.com (Z.C.); 18447053580@163.com (X.T.); niuxc@hanyao.com.cn (X.N.); baimeiting201027@163.com (M.B.); weibaihappy@126.com (W.B.); ruigangwang@126.com (R.W.); liguojing@imau.edu.cn (G.L.); 2 Plant Protection Institute, Inner Mongolia Academy of Agricultural and Animal Husbandry Sciences, Hohhot 010031, China

**Keywords:** PADRE, ABA, salt stress, DUF4228, ROS

## Abstract

The PADRE (Pathogen and abiotic stress response, cadmium tolerance, disordered region-containing) family of genes, which contains the structural DUF4228 domain of unknown function (DUF), has been reported to be associated with plant responses to abiotic stresses. However, the specific functions of this family in the salt stress response remain unknown. *AtPADRE13* is induced by salt stress and ABA (abscisic acid). After the overexpression of *AtPADRE13* in Arabidopsis, seeds were found to be insensitive to ABA treatment. After salt treatment, the overexpression lines presented a significantly lower survival rate, increased MDA (Malondialdehyde) content, and reduced antioxidant enzyme activities compared with the wild-type, and were more sensitive to salt stress. Transcriptome data analysis further revealed that *AtPADRE13* overexpression resulted in different degrees of down-regulation for a series of positive regulators related to ABA catabolism, transport, and their mediated plant responses to salt stress. In addition, the expression of genes related to ROS (reactive oxygen species) scavenging was down-regulated. In conclusion, AtPADRE13 plays a negative regulatory role in the response to salt stress in Arabidopsis.

## 1. Introduction

Soil salinization poses a significant challenge to global crop production, impacting an area of over 900 million hectares, one-third of which is dedicated to agriculture. This area is further impacted by human activities and climate change, leading to reduced agricultural productivity [1,2,3,4]. Salt stress is accompanied by osmotic stress, ionic toxicity, and oxidative stress, which harm all stages of plant growth and development, ultimately reducing crop yields [5,6]. Salt stress was demonstrated to reduce soil water potential and affect plant water and nutrient uptake, leading to changes in cell expansion pressure and stomatal closure, as well as impaired photosynthesis [7]. Prolonged salt stress leads to the accumulation of Na^+^ and Cl^−^, which affects K^+^ uptake and enzyme activities, disrupts chlorophyll synthesis, and reduces the photosynthetic rate [8,9]. Meanwhile, salt stress contributes to the rapid accumulation of ROS (reactive oxygen species), which triggers oxidative stress and damages intracellular macromolecules, leading to apoptosis in severe cases [10,11]. To cope with salt stress, plants adopt a number of strategies. Plants are able to sense changes in osmotic pressure in the soil [12] and maintain the intra- and extracellular water balance through the accumulation of osmoregulatory substances [13,14]. For ion homeostasis, plants exclude excess Na^+^ ions [8,15] and promote the uptake and transport of K^+^ ions [9,16] through specific ion channels and transporter proteins to maintain intracellular ion homeostasis. In response to oxidative stress, plants scavenge ROS through enzymatic and non-enzymatic antioxidant systems to protect cells from oxidative damage [17,18]. The synergy of these strategies is pivotal in facilitating the maintenance of normal physiological functions in plants and enhancing salt tolerance under salt-stressed environments.

Domains of unknown function (DUF) is a general term for a class of proteins with unknown functional structural domains included in the Pfam database [19,20]. With the development and maturation of histological technologies in recent years, the rate at which the functions of DUF proteins are resolved has increased. Many studies have shown that DUF proteins in plants are involved in a variety of important physiological and biochemical processes, such as the regulation of growth and development and responses to biotic and abiotic stresses. A number of these studies have shown that DUFs play an important role in the responses of plants to salt stress. For example, the overexpression of *GmCBSDUF3* enhanced tolerance to drought and salt stress in Arabidopsis [21]. The overexpression of *AhDGR2* (encoding a protein containing the DUF642 domain) from *Amaranthus hypochondriacus* L. in Arabidopsis reduced plant tolerance to salt stress [22]. Rice (*Oryza sativa* L.) *OsDSR2* (encoding a protein containing a DUF966 domain) is induced by a variety of abiotic stresses and phytohormones and is negatively regulated in rice in response to salt and drought stress [23]. In a previous study, the expression of *TaDUF966* in wheat (*Triticum aestivum*) was induced by salt stress, and *TaDUF966-9B* knockdown lines showed severe leaf curling under salt stress compared with that in the control [24]. OsSIDP366 (a DUF1644 family member) plays a negative regulatory role in response to salt and drought stress in rice [25].

The DUF4228 domain exists only in plants and has been the subject of a few functional studies. The overexpression of *MsDUF* in tobacco (*Nicotiana tabacum* L.) increased the sensitivity of tobacco seeds to osmosis and ABA (abscisic acid) [26]. Some genes in Arabidopsis can be induced by *Sclerotinia sclerotiorum*, and the proteins encoded by these genes all contain the DUF4228 structural domain. Subsequently, based on known functional information about the DUF4228 family, researchers have named these genes PADRE (Pathogen and abiotic stress response, cadmium tolerance, disordered region-containing) [27]. Different abiotic stresses induced the expression of multiple *AtDUF4228* genes in Arabidopsis [28]. In addition, *GmDUF4228* in soybeans (*Glycine max* L.) was induced by drought and salt stresses, and the overexpression of *GmDUF4228-70* improved tolerance to salt stress in soybeans [29]. *GhDUF4228* in cotton (*Gossypium hirsutum* L.) may be involved in abiotic stress responses, as silencing *GhDUF4228-67* reduced salt stress tolerance in cotton [30]. In summary, the PADRE family is related to plant responses to biotic and abiotic stresses, but the specific functions and implicated biological pathways remain to be studied in detail.

In this study, we found that overexpression of *AtPADRE13* resulted in seed insensitivity to ABA and reduced salt tolerance in seedlings, suggesting that this gene plays a negative regulatory role in salt tolerance in Arabidopsis. The results of this study may help to better understand the functions of the PADRE family, providing theoretical and practical guidance for improving the environmental adaptability of plants.

## 2. Results

### 2.1. Expression Pattern Analysis of AtPADRE13 Under Different Stresses

**Figure 1:**
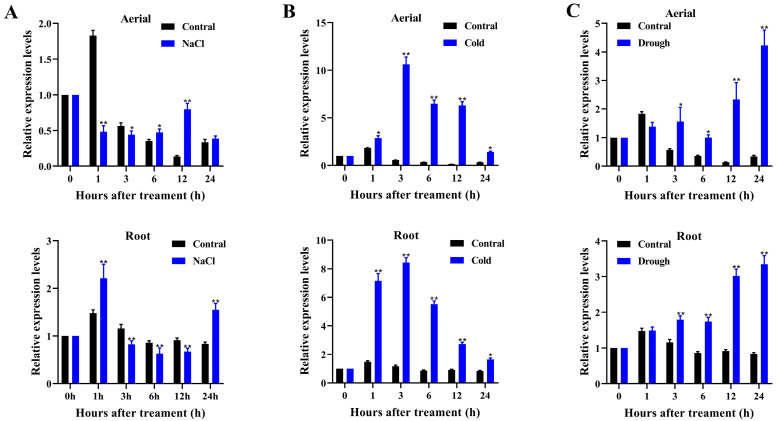
*AtPADRE13* expression pattern analysis. (**A**–**C**) *AtPADRE13* expression patterns under salt, cold, and drought stress. Error lines indicate ± SD of three technical replicates, * represents *p* < 0.05, and ** represents *p* < 0.01.

In a previous study, we identified 25 *AtPADRE* genes from Arabidopsis and analyzed the expression patterns of 16 of them (excluding *AtPADRE13*) under abiotic stress. Some of these genes were found to be significantly induced by abiotic stress [28]. Therefore, in this study, we named these 25 *AtPADRE* genes according to the chromosomal order (Table S2) and investigated the relevant biological functions of one of the members, *AtPADRE13*. According to previous studies, members of the *AtPADRE* family in Arabidopsis may be involved in plant responses to abiotic stresses. Therefore, we first analyzed the promoter sequence 2000 bp upstream of the *AtPADRE13* gene initiation codon (ATG) online using PlantCARE. The results demonstrated that this promoter region was enriched with multiple abiotic stress response elements (including four AREs associated with anaerobic responses, a low-temperature response element, and a drought-induced MYB-binding site) as well as multiple phytohormone response elements (three ABA-associated elements (ABREs), a methyl jasmonate response element (CGTCA), and an ethylene response element (ERE)) (Figure S1). It is reasonable to hypothesize that the *AtPADRE13* gene is subject to regulation by abiotic stresses as well as by phytohormone induction. Next, to further determine whether *AtPADRE13* was induced by abiotic stresses, we examined the expression levels of *AtPADRE13* in aerial parts and roots of Arabidopsis under osmotic, low-temperature, and salt stress, respectively, using RT-qPCR (Figure 1A–C). RT-qPCR analysis showed that the transcript level of *AtPADRE13* in the root was induced by salt stress and significantly up-regulated compared with that of the control. However, there was no significant change in the aerial (Figure 1A). Under cold or osmotic stress, the transcript levels of *AtPADRE13* were up-regulated in both the roots and aerial parts compared with the control (Figure 1B,C).

Taken together, *AtPADRE13* was clearly induced by a variety of abiotic stresses, and it is hypothesized that it is likely to be involved in the plant response to abiotic stresses.

### 2.2. Overexpression of AtPADRE13 Reduces Seed Sensitivity to ABA

Previous studies have shown that the *PADRE* gene family may be involved in ABA-mediated abiotic stress responses in plants [26]. The promoter element analysis described above also revealed the presence of an ABA-related element in the promoter region of *AtPADRE13*. Therefore, we also examined the expression pattern of *AtPADRE13* under ABA treatment. It was found that *AtPADRE13* was significantly induced in the wild type (Figure S2).

**Figure 2:**
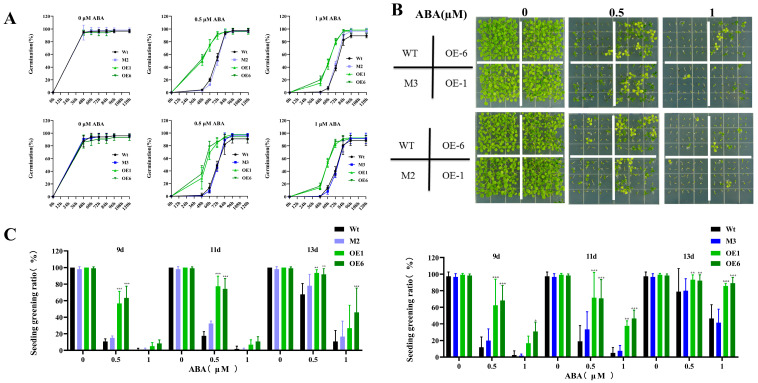
Phenotypes of OE lines, mutants, and wild-type seeds under ABA treatment. (**A**) Germination rate statistics. (**B**) Seed germination phenotypes. (**C**) Cotyledon greening rate statistics. Wild-type, OE lines, and mutants seeds were 30 seeds in each parallel, and medium containing different concentrations of ABA was set up in four parallels for calculation, and the errors represent ± SD values of the four parallels, * represents *p* < 0.05, ** represents *p* < 0.01, and *** represents *p* < 0.001.

To investigate whether *AtPADRE13* responds to ABA-mediated signaling pathways, we overexpressed *AtPADRE13* in Arabidopsis and selected two lines with high expression levels, OE-1 and OE-6 (Figure S3A,B). In addition, we used gene editing techniques to obtain two gene editing mutants, M2 and M3 (Figure S3C). Subsequently, OE lines, mutants, and wild types were tested under different concentrations of ABA treatments to determine their germination rate. In MS medium without added ABA, all seeds germinated normally with a germination rate of about 97%. In MS medium supplemented with ABA, the germination rates of all seed types decreased with an increase in ABA concentration. However, on this basis, the OE line had a significantly faster germination rate than that of the wild-type, whereas the rate of the mutant was not significantly different than that of the wild-type (Figure 2A). Meanwhile, the cotyledon greening rate also decreased significantly with an increase in ABA concentration. The greening rate of the OE lines was also still significantly higher than that of the wild-type, whereas that of the mutant was not significantly different from that of the wild-type (Figure 2B,C). These results suggest that *AtPADRE13* overexpression reduced the sensitivity of plants to ABA during the germination period.

### 2.3. Overexpression of AtPADRE13 Reduces Salt Stress Tolerance in Arabidopsis

**Figure 3:**
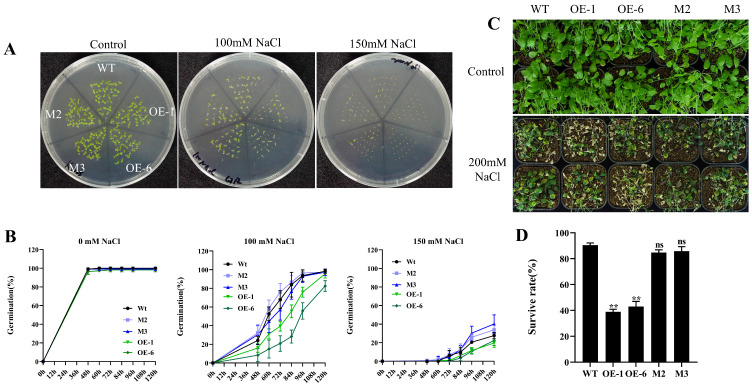
Phenotypes of OE lines, mutants, and wild-type plants under salt stress. (**A**) Seed germination phenotypes. (**B**) Germination rate statistics. Wild-type, OE lines, and mutant seeds were 28 seeds per parallel, and the medium containing different concentrations of NaCl was set up in four parallels for calculation, and the error represents the ±SD values of the four parallels. (**C**) Seedling phenotypes under salt stress. Twelve classes of plants per pot, and 24 plants each of wild-type, OE lines, and mutants were treated. The 4-week-old seedlings were watered with 200 mM NaCl solution every 5 days for 1 month. (**D**) Survival statistics. ** represents *p* < 0.01. ns indicates insignificant.

Expression pattern analysis showed that salt stress was able to induce the expression of the *AtPADRE13*, suggesting that this gene may be involved in plant responses to salt stress. To verify this result, we examined the germination rates of OE lines (OE-1\OE-6), gene-editing mutants (M2\M3), and wild-type seeds under treatments using different concentrations of NaCl. Under normal conditions, the germination rate of all seeds was close to 100%. However, the seed germination rate generally decreased with an increase in NaCl concentration, especially for the OE lines, which had a significantly lower germination rate than that of the wild-type, whereas the germination rate of the mutants was not significantly different from that of the wild-type (Figure 3A,B).

In further experiments, we treated 4-week-old plants with salt stress. Here, the OE lines exhibited severe premature senescence and leaf whitening, whereas mutants behaved similarly to the wild type without significant growth inhibition or premature senescence (Figure 3C). Survival statistics also showed that the survival rate of the wild-type was significantly higher than that of the OE lines (twice as high), while the survival rate of the mutants was not significantly different from that of the wild-type (Figure 3D).

**Figure 4:**
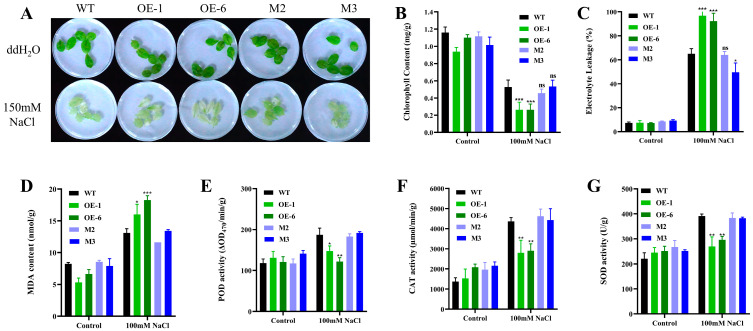
Phenotypes and physiological indices of OE lines, mutants, and wild-type plants under salt stress in isolated leaves. (**A**) Phenotypes of isolated leaves under salt treatment. Rosette leaves of 4-week-old seedlings were treated in 100 mM NaCl solution for 36 h. (**B**) Chlorophyll content. (**C**) Electrolyte leakage rate. (**D**) Malondialdehyde (MDA) content. (**E**) Peroxidase (POD) activity. (**F**) Catalase (CAT) activity. (**G**) Superoxide dismutase (SOD) activity. Errors represent 3 parallel ± SD values, * represents *p* < 0.05, ** represents *p* < 0.01, and *** represents *p* < 0.001, ns indicates insignificant.

We also treated isolated leaves with salt stress and found that the OE lines had more severe leaf bleaching than the wild-type (Figure 4A). Analysis of physiological indicators showed that the OE lines had significantly reduced chlorophyll content (Figure 4B) and more electrolyte leakage (Figure 4C). In terms of oxidative stress, malondialdehyde (MDA) content was increased in the OE lines (Figure 4D). However, the antioxidant enzyme activities, including those of peroxidase (POD), catalase (CAT) and superoxide dismutase (SOD), were lower than the activities of the wild-type (Figure 4E–G).

Taken together, the overexpression of *AtPADRE13* may result in plants that are more sensitive to salt stress, as evidenced by reduced seed germination, impeded plant growth, decreased survival, and weakened antioxidant capacity. However, the germination rate, growth status, and survival rate of the gene editing mutant under salt stress were not significantly different from those of the wild-type, suggesting that the mutation of *AtPADRE13* may not affect the salt tolerance of the plant, possibly due to the redundancy of its gene functions. Based on this speculation and in conjunction with our previous study, AtPADRE13 and AtPADRE21 clustered together in a phylogenetic analysis [28]. We compared the protein sequences of AtPADRE13 and AtPADRE21 and found 78.69% identity (Figure S4). This result further suggests that there may be redundancy in the related gene functions, but further verification is needed.

### 2.4. Comparative Transcriptome Analysis of the Arabidopsis Wild-Type and OE Lines

**Figure 5:**
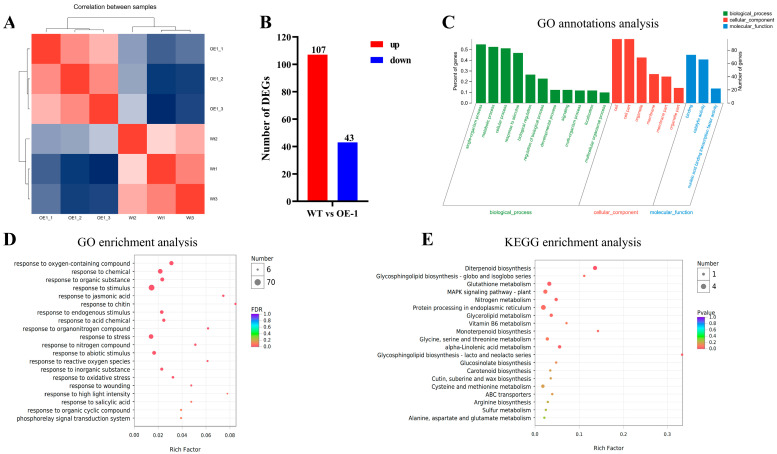
Transcriptome data analysis of wild-type and OE-1. (**A**) Inter-sample correlation analysis. (**B**) Differential gene number counts. (**C**) Differential gene GO classification analysis. The horizontal coordinate indicates the secondary classification of GO, the left vertical coordinate indicates the percentage of genes/transcripts included in this secondary classification to the total number, the right vertical coordinate indicates the number of genes/transcripts comparing on this secondary classification, and the three colors indicate the three major branches of GO (i.e., BP, CC, and MF). (**D**) Differential gene GO enrichment analysis. The vertical axis indicates GO Term, and the horizontal axis indicates Rich factor, i.e., the ratio of the number of genes/transcripts enriched in the GO term to the number of annotated genes/transcripts. (**E**) Differential gene KEGG enrichment analysis. Vertical axis indicates the pathway name, and the horizontal axis indicates the Rich factor.

To further explore the mechanism underlying the role of *AtPADRE13* in plant salt tolerance, we performed an in-depth analysis of the transcriptomes of Arabidopsis wild-type and OE-1. Cluster analysis among samples showed that the expression of genes among different samples could clearly distinguish between the wild-type and OE-1 (Figure 5A). Differential gene analysis identified 303 genes with significant changes in expression, including 107 up-regulated and 43 down-regulated genes (Figure 5B). GO functional classification categorizes the differential genes into three groups: biological processes, cellular components, and molecular functions. The analysis revealed that the differential genes were mainly focused on biological processes (especially metabolism and responses to stimuli), cellular components (including organelles and membranes), and molecular functions (mainly binding and catalytic activities) (Figure 5C). GO enrichment analysis showed that the differential genes were mainly associated with response processes such as those related to chitin, methyl jasmonate, nitrogen compounds, and reactive oxygen species. The differential genes were also significantly enriched in response processes such as stimulation, biotic stress, and abiotic stress (Figure 5D). KEGG enrichment analysis revealed that the differential genes were mainly enriched in processes such as diterpene biosynthesis, monoterpene biosynthesis, and the glycosphingolipid biosynthesis-lactose and neo-lactose series. These genes were also significantly enriched in the plant MAPK signaling pathway (Figure 5E).

The results showed that the overexpression of *AtPADRE13* significantly affected gene expression patterns in Arabidopsis, especially in genes related to metabolic processes, responses to stimuli, and antioxidant defense. These changes may be related to the function of *AtPADRE13* in plant response to salt stress. The overexpression of *AtPADRE13* may result in plants becoming more sensitive to salt stress, possibly because it affects relevant metabolic processes and antioxidant defense mechanisms. In addition, *AtPADRE13* may regulate plant responses to abiotic stress by affecting the MAPK signaling pathway. These findings provide important insights to build upon in future studies on the mechanism of the role of *AtPADRE13* in plant salt tolerance.

### 2.5. AtPADRE13 Overexpression Negatively Regulates the Expression of Salt Stress-Related Genes

**Figure 6:**
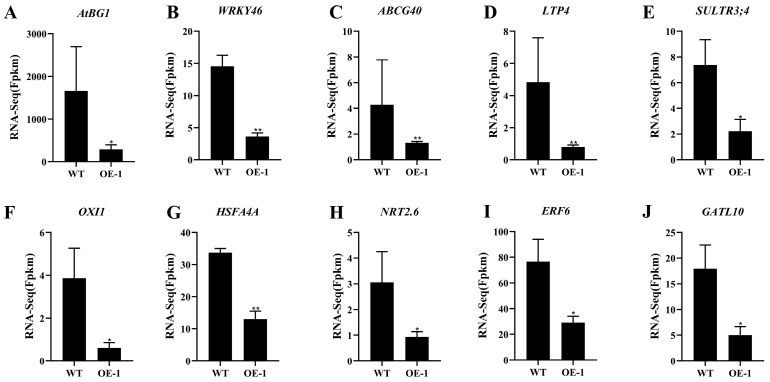
Changes in expression of related genes in the transcriptome. * represents *p* < 0.05, and ** represents *p* < 0.01.

By further mining the different genes, we found that the expression of some genes related to ABA-mediated plant responses to salt stress was significantly changed in the OE lines compared with that in the wild-type. For example, *AtBG1* encodes a β-glucosidase that rapidly converts ABA-GE (ABA-glucose) to active ABA, thereby increasing the level of ABA in the plant and improving plant resistance. Transcriptome data analysis revealed that *AtBG1* was significantly down-regulated in the *AtPADRE13* overexpression material, and it was hypothesized that *AtPADRE13* may affect *AtBG1* and thus be involved in influencing ABA-mediated plant tolerance to salt stress (Figure 6A). *WRKY46* expression was repressed by ABA and up-regulated after salt stress. Additionally, the mutation reduced plant salt tolerance, which was down-regulated in the *AtPADRE13* overexpression material (Figure 6B). AtABCG40, a member of the ABC transporter protein family, is located on the plasma membrane and is responsible for ABA uptake. The mutation of this protein reduces plant salt tolerance. *AtABCG40* expression was down-regulated through the overexpression of *AtPADRE13* (Figure 6C). Lipid transfer protein 4 (*LTP4*) was up-regulated by ABA, salt, and drought stress, but significantly down-regulated after *AtPADRE13* overexpression (Figure 6D). SULTR3;4 is a sulfate transporter protein that transports sulfate to chloroplasts. *SULTR3;4* mutants were found to reduce ABA levels and are more sensitive to salt stress. In addition, *SULTR3;4* expression was down-regulated upon the overexpression of *AtPADRE13* (Figure 6E). Taken together, *AtPADRE13* overexpression resulted in the down-regulation of ABA-related positive regulators, which are hypothesized to negatively regulate ABA-mediated responses to salt stress.

The expression of some genes related to oxidative stress signaling and ROS scavenging among the differential genes also showed significant changes. For example, oxidative signal-inducible kinase (OXI1) mediates oxidative stress signaling in plants. Changes in OXI1 activity are critical for mitogen-activated protein kinase (MAPK)-mediated responses such as MPK3 and MPK6. The overexpression of *AtPADRE13* resulted in the down-regulation of *OXI1* gene expression (Figure 6F). HSFA4A is a heat shock transcription factor and acts as a substrate for MPK3/MPK6 oxidative stress signaling. The knockdown of HSFA4A resulted in salt stress sensitivity in Arabidopsis, and the overexpression of *HSFA4A* altered the transcription of genes regulated by oxidative stress. Correspondingly, *HSFA4A* was significantly down-regulated after *AtPADRE13* overexpression (Figure 6G). The high-affinity nitrate transporter protein (NRT2.6), whose activity was negatively correlated with ROS accumulation, and the expression of the gene encoding this protein were significantly down-regulated in the *AtPADRE13* overexpression material (Figure 6H). Ethylene response element binding factor 6 (ERF6) plays an important role as an antioxidant regulator in plant responses to abiotic stress; this gene was significantly down-regulated in the overexpression material (Figure 6I). Galactosyltransferase-like 10 (GATL10), also known as galactitol synthase, is a key enzyme in the synthesis of raffinose. This enzyme helps scavenge hydroxyl radicals and protects plant cells from salt- or cold-induced oxidative damage. *GATL10* gene expression was significantly down-regulated in the overexpression material (Figure 6J).

## 3. Discussion

There are few reports on the PADRE family, all of which suggest that this family is associated with plant responses to abiotic stress. Previous promoter analyses of *MsDUF* [26], *GmDUF4228-70* [29], and *GhDUF4228-67* [30] found them to contain a variety of cis-acting elements associated with abiotic stresses, in addition to an ABA response element. Our previous study conducted a phylogenetic analysis of 489 DUF4228 proteins from 14 land plant species and found the DUF4228 domain to be highly conserved. We also analyzed the upstream cis-acting elements of 25 *ATDUF4228* genes in Arabidopsis that respond to abiotic stress and hormones. In addition, we analyzed the expression patterns of 16 *AtDUF4228* genes under abiotic stress. The results indicate that some of these genes may be involved in plant stress resistance pathways [28]. The analysis of the *AtPADRE13* promoter region in this study similarly identified elements associated with responses to abiotic stresses and phytohormones (Figure S1). On the other hand, expression pattern analysis under abiotic stress also revealed that *AtPADRE13* was significantly up-regulated after drought, salt, cold, and ABA treatments (Figure 1A–C and Figure S2). In conclusion, *AtPADRE13* is associated with plant responses to abiotic stress and may also be involved in responses mediated by the phytohormone ABA.

The overexpression of *MsDUF* in tobacco enhanced tolerance to drought and salt stress in transgenic plants. Similarly, the overexpression of *GmDUF4228-70* in soybean was found to significantly enhance tolerance to drought and salt stress [29]. Silencing of *GhDUF4228-67* yielded decreased tolerance to salt stress in cotton, suggesting that GhDUF4228-67 is a positive regulator of cotton’s salt stress response [30]. The tolerance of transgenic plants to salt stress was increased to different degrees after overexpression of the above genes, which played positive regulatory roles in plant responses to salt stress. In this study, however, the overexpression of *AtPADRE13* resulted in plants that were more sensitive to salt stress treatments (Figure 3) and played a negative regulatory role in the response of Arabidopsis to salt stress. These results conflict with the functions of the previously studied gene. Analyzing the evolutionary tree in the above article provided the following information. AtPADRE13, MsDUF, and GmDUF4228-70 were each located in three different groups in the evolutionary tree [29], while AtPADRE13 and GhDUF4228-67 were each located in two groups [30]. This result suggests that the functions of different subgroups of PADRE in plant responses to abiotic stress are not entirely consistent, and that PADRE’s functions are diverse in different plants and groups.

The results of the phenotypic data and transcriptome analysis demonstrate that the sensitivity of *AtPADRE13* overexpressing lines to salt stress is closely related to the systematic alteration of their gene expression patterns. GO and KEGG enrichment analyses showed that differential genes were mainly enriched in diterpene and monoterpene biosynthesis, stimulus responses, antioxidant defense, and the MAPK signaling pathways (Figure 5), suggesting that metabolic reprogramming and signaling defects may collectively hinder the activation of adaptive defenses, resulting in growth inhibition and reduced survival in OE lines under salt stress (Figure 3).

As demonstrated in previous studies, the expression of *MsDUF* in tobacco was found to result in increased seed sensitivity to ABA, elevated levels of ABA, and the up-regulation of genes involved in ABA synthesis [26]. However, the present study demonstrated that *AtPADRE13* overexpression led to a reduction in seed sensitivity to ABA (Figure 2). Transcriptomic data demonstrated that AtBG1, a key component in the activation of ABA [31], exhibited a substantial decrease in expression in the overexpressing lines (Figure 6A). This decline may have reduced ABA levels, potentially leading to impairment in the ABA-mediated stress response. Concurrently, AtABCG40, a member of the ABC transporter family of proteins situated at the plasma membrane and responsible for ABA uptake [32], exhibited a significant decrease in expression (Figure 6C). Therefore, it was hypothesized that the expression of *AtPADRE13* might interfere with ABA metabolism and transport, thereby reducing intracellular ABA levels and resulting in plant hypersensitivity to salt stress. Furthermore, several key ABA-mediated salt stress response genes exhibited a decrease in expression in the overexpressing lines (Figure 6). These genes include *WRKY46*, whose mutation was shown to reduce the salt tolerance of the plant and is both repressed by ABA and up-regulated after salt stress [33] and SULTR3;4, a sulfate transporter protein that transports sulfate to the chloroplasts, whose mutants have reduced ABA levels and are more sensitive to salt stress [34]. These genes play pivotal roles in enhancing salt stress tolerance in plants, and their repressed expression may impair ABA signaling and weaken salt stress responses.

In the context of salt stress, plants exhibit an accelerated and substantial accumulation of ROS, which can lead to a state of redox imbalance and severe oxidative stress [10]. Plants typically employ enzymatic antioxidant systems (SOD, POD, CAT, etc.) to scavenge ROS and maintain homeostasis [17]. In this study, we found that the SOD, POD, and CAT activities of *AtPADRE13* overexpressing lines were lower than those of the wild-type after experiencing salt stress (Figure 4E–G), suggesting that their ROS scavenging ability was impaired. Transcriptome mining revealed that genes implicated in oxidative stress signaling and ROS scavenging exhibited a substantial decrease in expression in the overexpressed material. OXI1, which functions as a mediator of oxidative stress signaling and is imperative for MAPK response [35]; HSFA4A, a substrate of MPK3/MPK6 oxidative stress signaling whose knockdown renders Arabidopsis more susceptible to salt stress and whose overexpression alters the transcription of related genes [36]; NRT2.6, whose activity exhibits a negative correlation with ROS accumulation [37]; the antioxidant regulator ERF6 [38]; GATL10, the key enzyme for cotton seed sugar synthesis, which scavenges hydroxyl radicals and protects cells from salt- or cold-induced oxidative damage [39].

**Figure 7:**
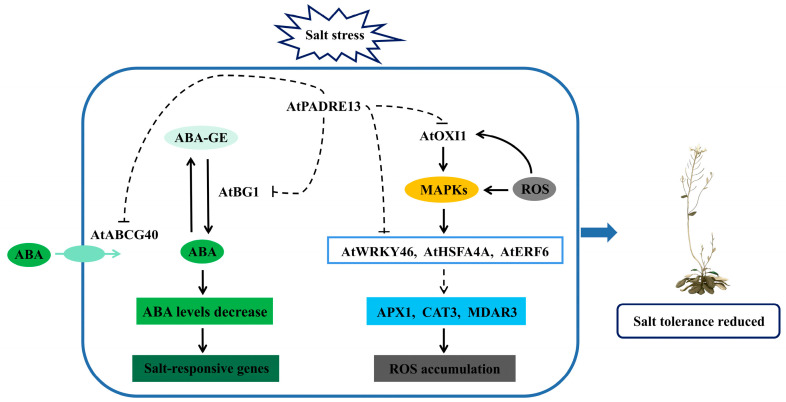
Modeling of AtPADRE13 negative regulation of Arabidopsis response to salt stress.

In summary, by integrating phenotypic and transcriptomic analyses and delving into existing literature on ABA and salt stress [40,41,42,43,44], we have preliminarily established a working model for AtPADRE13’s response to salt stress (Figure 7).

Overexpression of *AtPADRE13* enhances Arabidopsis’s sensitivity to salt stress and ABA. It likely affects AtBCG40-mediated ABA transport into cells and AtBG1-mediated conversion of inactive ABA-GE to active ABA, reducing intracellular ABA levels. Additionally, AtPADRE13 influences MAPK-mediated AtWRKY46, AtHSFA4A and AtERF6 activities, affecting ROS -clearance -related gene expression (*APX1*, *CAT3*, *MDAR3*), and may impact ROS-activated AtOXI1, leading to ROS accumulation. In summary, AtPADRE13 may reduce salt stress tolerance by lowering ABA levels and inhibiting ROS clearance. However, further research on the direct targets of AtPADRE13 is required to refine the salt stress regulation model.

## 4. Materials and Methods

### 4.1. Plant Growth and Treatment Conditions

The wild-type Arabidopsis thaliana used in this study was Columbia type 0. Plants were grown at 22 °C, and the relative humidity was generally controlled at 60–70%. LED tubes (Purchased from Sanan Optoelectronics Co., Ltd., Wuhu, China) were used to provide the light source, with the light intensity controlled at 120–150 μM/(m^2^·s) and a light-dark cycle of 16/8 h.

Germination experiments: Wild-type, overexpressed and mutant seeds were sterilized and sown in a 1/2 MS solid medium containing different concentrations of ABA (Purchased from Coolaber Technology Co., Ltd. in Beijing, China) (0.5 µM and 1 µM) or NaCl (purchased from Tianjin Third Factory of Chemical Reagents in Tianjin, China) (100 mM and 150 mM). Germination was counted every 12 h, and cotyledon greening was counted on days 9, 11, and 13.

Seedling salt treatment: 4-week-old seedlings were watered with 200 mM NaCl every 5 days, and the survival rate was counted, photographed, and recorded after one month of treatment.

Salt treatment of isolated leaves: Rosette leaves of 4-week-old seedlings were treated with 100 mM NaCl solution for 36 h and then photographed and recorded. Next, samples were collected for physiological indexing.

Expression pattern analysis under abiotic stress: Abiotic stress treatments were modeled after those in previous studies [28]. ABA treatment: Arabidopsis seedlings grown normally on a 1/2MS solid medium for 2 weeks were sampled after different treatment durations (0, 1, 3, 6, 12, 24 h) using a 1/2MS liquid medium containing 100 μM ABA. RNA was extracted for reverse transcription to synthesize cDNA using the LightCycler 480 SYBR Green I Master kit. (Purchased from Roche Pharmaceutical Co., Ltd. in Shanghai, China) All samples contained three technical replicates, and the relative expression of the *AtPADRE13* gene was calculated according to the 2^−ΔΔCT^ method.

### 4.2. Acquisition of Transgenic Material

To obtain *AtPADRE13* OE lines, the ORF fragment of *AtPADRE13* was amplified via PCR using Arabidopsis cDNA and ligated into a pCanG-HA vector. The CRIPSR-Pv2.0 website was utilized to design the sgRNA, and the amplified target fragment was ligated to the pHEE401E vector using Golden Gate [45]. The recombinant vector was transferred into Agrobacterium tumefaciens GV3101, and wild-type Arabidopsis was transformed using the flower dip method [46]. The primers used in this study are shown in Table S1.

### 4.3. Physiological Indicator Tests

We assessed the changes in peroxidase (CAT, POD, SOD) activities and the MDA content in leaves after salt stress using kits (Grace, Suzhou, China) and calculated the result by reading absorbance values at 510, 470, 450, 532, and 600 nm using a microplate reader [47]. The chlorophyll content and electrolyte leakage rate were determined in accordance with the methods used in previous studies [48]. For the former analysis, salt-treated leaves were taken, and the extract was mixed with acetone and anhydrous ethanol (purchased from Tianjin Third Factory of Chemical Reagents in Tianjin, China) in equal proportions. Next, the absorbance values at 645 nm and 663 nm were measured and calculated by substituting into the formula. For the latter analysis, the leaves were taken, and 6 mL of deionized water was added. This mixture was then oscillated for 3 h, and the electrical conductivity was measured before and after boiling by substituting the values into the formula for calculations.

### 4.4. RNA-Seq and Data Analysis

RNA was extracted from rosette leaves of 20-day *AtPADRE13* OE-1 and wild-type, reverse transcribed, and sequenced on the HiSeq xten/NovaSeq 6000 platform at Majorbio in Shanghai. Differentially expressed genes were analyzed using the DESeq2 software [49]. GO enrichment [50] and KEGG pathway enrichment [51] were performed on differentially expressed genes.

### 4.5. Gene Expression Analysis

Total RNA was extracted using a Plant RNA Extraction Kit (TIANGEN, Beijing, China), and cDNA was later synthesized using a Reverse Transcription Kit (TaKaRa, Beijing, China). RT-qPCR was performed using a SYBR Green I Master Mix Kit (TaKaRa, Beijing, China). The reaction program was 95 °C for 2 m, with 40 cycles performed for 5 s at 95 °C and for 10 s at 60 °C. The relative RNA transcript levels of genes were calculated using the 2^−ΔΔCT^ method. AtEF1α (*At5G60390*) was used as a reference gene [52].

## Data Availability

RNA-seq raw datasets generated during the current study have been deposited in the NCBI repository under BioProject code PRJNA1245673. The datasets that support the conclusions of this paper are included in this paper and its additional files.

## Supplementary Materials

The following supporting information can be downloaded at: https://www.mdpi.com/article/10.3390/plants14101514/s1, Figure S1: *AtPADRE13* promoter cis-acting element analysis. Figure S2. *ATPADRE13* gene expression under ABA treatment. Figure S3. *AtPADRE13* Acquisition of OE lines and gene-editing mutants. (A) Gene cloning and expression vector construction. (B) OE lines expression assay. (C) *AtPADRE13* gene structure and sgRNA target location. Figure S4: AtPADRE13 and AtPADRE21 protein sequence comparison. Table S1: The primers used in this study. Table S2: Gene name of *AtPADRE*.
