# Supplementary material for: AtPADRE13 Negatively Regulates Salt Stress Tolerance in Arabidopsis thaliana
Source: Plants (Basel). 2025 May 19;14(10):1514. doi: 10.3390/plants14101514 (PMC12114685; doi:10.3390/plants14101514)

AtPADER13 negatively regulates the response of Arabidopsis thaliana to salt stress

Supplementary Material

Supplementary Table S1 The primers used in this study

Primer name	Sequence (5, -3,)
HA-AtPADRE13-F	CGCCTCCCTCGTCGACATGGGCAACTACGTTTCATG
HA-AtPADRE13-R	GATCGGGGAAATTCGAGCTCTCGACTAATGTCTCCTTCCGT
QRT-AtPADRE13-F	CCGACATGGCTAGGCTCTACCTC
QRT-AtPADRE13-R	CGCCGAGAACTCTTCTATGTCTTCAA
Oligo-F	ATTGAAAACGTCATCGTCACCGT
Oligo-R	AAACACGGTGACGATGACGTTTT

Supplementary Table S2 Gene name of *AtPADRE*

Locus ID	Gene Name
At1g06980	*AtPADRE1*
At1g10530	*AtPADRE2*
At1g18290	*AtPADRE3*
At1g21010	*AtPADRE4*
At1g28190	*AtPADRE5*
At1g29195	*AtPADRE6*
At1g60010	*AtPADRE7*
At1g64700	*AtPADRE8*
At1g71015	*AtPADRE9*
At1g76600	*AtPADRE10*
At2g23690	*AtPADRE11*
At2g30230	*AtPADRE12*
At3g03280	*AtPADRE13*
At3g10120	*AtPADRE14*
At3g50800	*AtPADRE15*
At3g61920	*AtPADRE16*
At4g02090	*AtPADRE17*
At4g37240	*AtPADRE18*
At5g03890	*AtPADRE19*
At5g12340	*AtPADRE20*
At5g17350	*AtPADRE21*

At5g50090	*AtPADRE22*
At5g62900	*AtPADRE23*
At5g66580	*AtPADRE24*
At5g67620	*AtPADRE25*

Supplementary Figures S1. *AtPADRE13* promoter cis-acting element analysis.

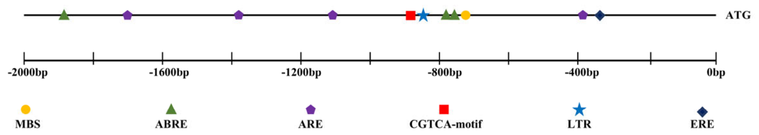

Supplementary Figures S2. *ATPADRE13* gene expression under ABA treatment.

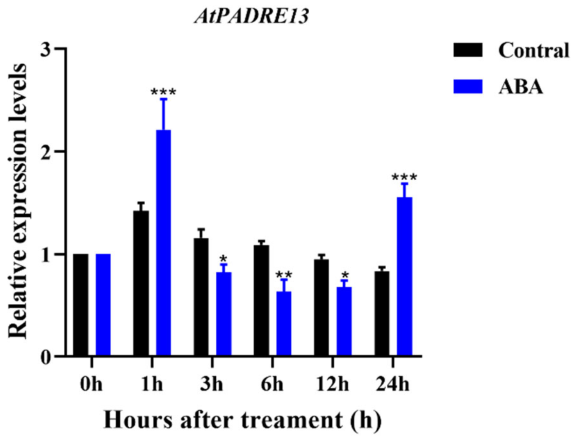

Supplementary Figures S3. *AtPADRE13* Acquisition of OE lines and gene-editing mutants. (A) Gene cloning and expression vector construction. (B) OE lines expression assay. (C) *AtPADRE13* gene structure and sgRNA target location.

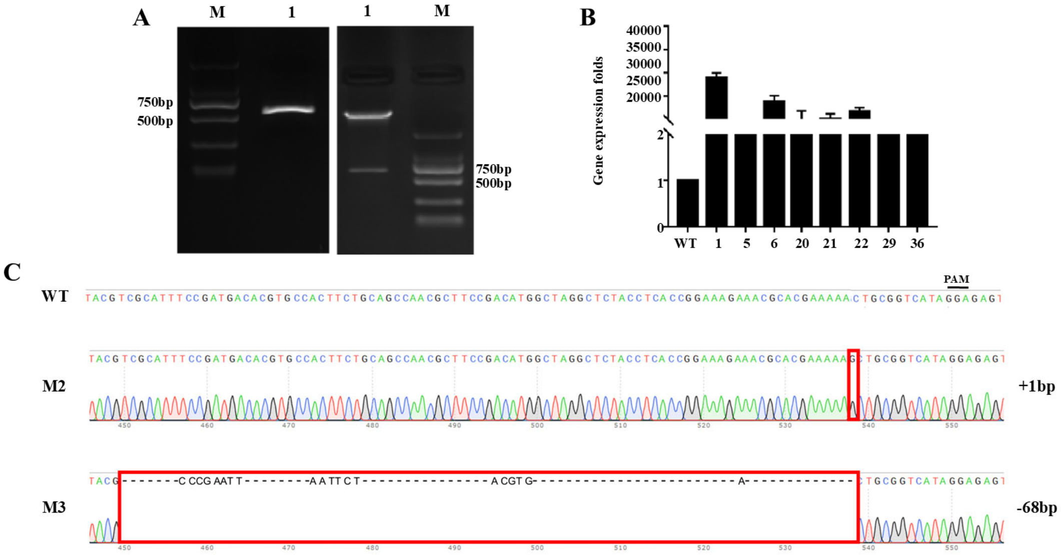

Supplementary Figures S4. AtPADRE13 and AtPADRE21 protein sequence comparison.

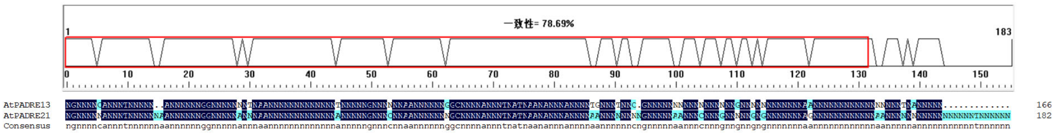

Supplement: Supplementary file 1 [file plants-14-01514-s001.zip › plants-3601352-supplementary.pdf]
